# Application of the screening and indirect cohort methods to evaluate the effectiveness of pneumococcal vaccination program in adults 75 years and older in Taiwan

**DOI:** 10.1186/s12879-020-05721-0

**Published:** 2021-01-10

**Authors:** Wei-Ju Su, Pei-Hung Chuang, Luan-Yin Chang, Hsiu-Yun Lo, Chuen-Sheue Chiang, Ez-Tzu Wang, Chin-Hui Yang

**Affiliations:** 1 Division of Acute Infectious Diseases, Centers for Disease Control, Ministry of Health and Welfare, 6, Linsen South Road, Taipei, 100 Taiwan, Republic of China; 2 Department of Pediatrics, National Taiwan University Hospital, Taipei, Taiwan, Republic of China; 3 Center for Prevention and Treatment of Occupational Injury and Diseases, Taipei Veterans General Hospital, Taipei, Taiwan, Republic of China; 4 Division of Clinical Toxicology and Occupational Medicine, Department of Medicine, Taipei Veterans General Hospital, Taipei, Taiwan, Republic of China

**Keywords:** Pneumococcal polysaccharide vaccines, Vaccine effectiveness, Invasive pneumococcal disease, Screening method, Indirect cohort method

## Abstract

**Background:**

The Taiwanese national 23-valent pneumococcal polysaccharide vaccine (PPV23) program in adults ≥75 years of age and the 13-valent pneumococcal conjugate vaccine (PCV13) program for children were implemented in 2008 and 2013, respectively. In this study we evaluated PPV23 vaccine effectiveness (PPV23VE) in the elderly, with regard to both direct protection from the vaccine itself and the indirect protection conferred by PCV13 immunization in children.

**Methods:**

The incidence of invasive pneumococcal disease (IPD) in Taiwan from July 2008 to June 2016 was collected from IPD surveillance data. A comparison of IPD incidence with a nationwide vaccination registry allowed an estimation of PPV23VE by the screening and indirect cohort methods.

**Results:**

The incidence of IPD in adults ≥75 years of age ranged from 13.9 per 100,000 inhabitants during the period July 2008–June 2013 to 10.4 per 100,000 inhabitants between July 2013 and June 2016 (relative risk [RR]: 0.75; 95% confidence interval [95% CI]: 0.67–0.85). According to the screening method, PPV23VE against death within 30 days of IPD onset, all IPD, and PPV23-serotype IPD was 32.5% (95% CI: 17.5–44.7%), 33.9% (95% CI: 25.2–41.5%) and 43.4% (95% CI: 34.4–51.2%), respectively. PPV23VE with the indirect cohort method was 39.0% (95% CI: 15.5–55.9%) for all PPV23 serotypes and 71.5% (95% CI: 44.2–85.4%) for 11 serotypes included in PPV23 but not in PCV13. During the period July 2008–June 2012, PPV23VE against PPV23-serotype IPD was 55.1% (95% CI: 27.2–72.3%).

**Conclusions:**

PPV23 is able to prevent IPD and 30-day fatality in adults 75 years of age and older due to a combination of direct effects from PPV23 and indirect effects from PCV13. It might confer higher protection against PPV23-serotype IPD before the introduction of PCV13 program in children.

**Supplementary Information:**

The online version contains supplementary material available at 10.1186/s12879-020-05721-0.

## Background

Invasive pneumococcal disease (IPD), defined as the isolation of *S. pneumoniae* from a normally sterile body site, such as the blood, cerebrospinal fluid or pleural effusion, poses significant health threats to young children (< 5 years of age), older adults, and individuals with chronic medical conditions [1, 2]. In the USA, a national pneumococcal conjugate vaccine (PCV) program for children was introduced in 2000, which was earlier than most countries worldwide [3]. The pediatric population was chosen because the incidence of IPD was higher in children under the age of 5 years (71.8 per 100,000 inhabitants) than in adults ≥ 65 years (57.6 per 100,000 inhabitants) [4]. However, by 2016, the main disease burden of IPD in the USA had shifted from children < 5 years (8 per 100,000 inhabitants) to adults ≥ 65 years (24 per 100,000 inhabitants) [5]. Similar findings were determined in Taiwan from the pre- to the post-PCV immunization era [3, 6]. Age-related changes in IPD epidemiology over time indicate the need to evaluate the impact of pneumococcal immunization programs and vaccine effectiveness (VE) both in children and in older adults [3, 7, 8].

National 23-valent pneumococcal polysaccharide vaccine (PPV23) and 13-valent PCV (PCV13) vaccination programs were started in Taiwan in 2008 and 2013, respectively [3, 6, 9]. Thus, any evaluation of the protective effects of PPV23 in the elderly must take into account both direct protection from PPV23 vaccination and indirect protection from PCV13 immunization in children [10].

While evidence-based recommendations form the cornerstone of the vaccination policy of the World Health Organization (WHO) and of many developed countries, such data are scarce in Africa and Asia [7, 11, 12]. Available systematic reviews and meta-analyses have concluded that PPV23 confers protection against IPD but the duration of protection is unclear [11, 13–16]. We therefore investigated the PPV23 program in older adults in Taiwan over a study period of 8 years, from the pre- to the post-PCV13 immunization era.

## Methods

### The PPV23 vaccination program and vaccine coverage

A national PPV23 vaccination program aimed at the elderly (≥75 years), with vaccines donated by the Formosa Plastic Group, was implemented in 2008 [9]. In addition, during the study period seven counties/cities (Tainan, Yunlin, Taichung, and Lianjiang counties, and the cities of Taichung, Tainan, and Chiayi) introduced local, publicly funded PPV23 vaccination programs that provided free PPV23 immunization to residents 65–74 years of age.

### Data sources

Data on IPD cases in patients of all ages in whom disease onset was between July 1, 2008, and June 30, 2016, were obtained from the national IPD surveillance system, a hospital laboratory- and case-based passive surveillance system for monitoring IPD established on October 15, 2007, by the Taiwan Centers for Disease Control [2, 17]. The IPD surveillance database contains demographic and clinical data, including IPD onset date and high-risk medical conditions (HRMCs, such as immunodeficiency/cancer, chronic obstructive pulmonary disease, congenital heart disease, splenectomy/asplenism, neurological disease, organ transplantation, congenital metabolic disorders, and other major illnesses) [2].

The PPV23 vaccination date was obtained from the National Immunization Information System (NIIS) database, described in previous studies [2, 18, 19]. The national vaccine registry is primarily designed to collate childhood vaccination data into a single web-based repository. However, it has been extended to include the registration of publicly funded PPV23 vaccination in the elderly and voluntary, self-paid childhood and adult vaccination. By linking the NIIS to the National Household Registration System, which includes all citizens with identifiers, we were able to calculate the coverage rate of PPV23 immunization in Taiwan for vaccine- targeted age groups.

### Statistical analysis

Incidence rates of IPD were calculated per 100,000 inhabitants, and specific incidence rates by age groups (≤5, 6–64, 65–74, and ≥75 years) and vaccine serotype. Incidence rate of serotype 19A-IPD was specifically characterized because it emerged after 7-valent PCV introduction and tends to result in more complicated pneumonia with empyema [6]. The rates were compared using relative risk (RR) and the 95% confidence interval (95% CI). Age-specific data on inhabitants in Taiwan were obtained from the Taiwan National Household Registration on a yearly basis. The Cochran-Armitage test was used to assess the trends in annual IPD incidence. Individuals were considered to be vaccinated if their PPV23 vaccination date was ≥14 days before IPD onset. We excluded those received neither 2 doses of PPV23 nor PCV13 plus PPV23 from the study. Differences between vaccinated and unvaccinated patients were estimated using a chi-squared test or Fisher’s exact test to compare proportions and by a linear regression model to compare continuous variables.

PPV23VE was calculated using two methods: the screening method and the indirect cohort (Broome) method. The screening method, described by Farrington [10, 20, 21], is based on the comparison of the proportion of vaccinated cases with the proportion of the vaccinated population [21], and by its assumption in nature, instead of data on a control group, data on the whole population are used for contrast with vaccine coverage in the cases [22]. The VE was expressed as $\mathrm{VE}=\frac{1-\left[ Pc\left(1- Pp\right)\right]}{Pp\left(1- Pc\right)}\ast 100\%$, where *Pc*=the proportion of cases who have been vaccinated and *Pp* =the proportion of the target population who have been vaccinated. Using logistic regression models, VE obtained by the screening method could control for confounding variables of age group (75–84 and all 85+) and sex when data on the vaccination coverage in each subgroup was available.

On the other hand, the indirect (Broome) cohort design used IPD cases caused by PPV23 vaccine types (VT) as the case group and IPD cases caused by non-PPV23 serotypes (ST) as the control group (non-cases, the comparison group) [10, 23]. The basic assumption in the indirect cohort design is that PPV23 vaccine provides no protection against and does not increase the risk of IPD caused by non-PPV23 ST. [10, 23–25]. VE was estimated by comparing the vaccination odds (compared to no PPV23 vaccination) of cases with controls and calculated as (1− odds ratio) × 100% [10, 23]. Potential confounders, including sex, age, HRMC, and year of symptom onset, were adjusted by logistic regression. The statistical power of indirect cohort method would decrease as the vaccine coverage increases (> 50%) and fewer VT cases occur [23]. VE was also estimated for IPD caused by 1) 11 serotypes included in PPV23, but not found in PCV13 (PPV23-non PCV13 VT); 2) each serotype included in PPV23 that had been identified in at least 30 cases, in which other PPV23 vaccine serotypes were excluded for analysis. VE in preventing PPV23-serotype IPD, calculated according to the indirect cohort method, was also expressed as the number needed to vaccinate per case prevented [10, 24]. To estimate VE according to different intervals after PPV23 vaccination, the interval between IPD onset and the date of vaccination was categorized as ≤ 1 year, > 1 and ≤2 years, > 2 and ≤ 3 years, > 3 and ≤ 4 years, > 4 and ≤5 years, and ≥ 5 years. All analyses were conducted using SAS software (ver. 9.4; SAS Institute, Cary, NC, USA).

### Ethical statement

The Taiwan CDC approved the protocol of this study and waived the requirement for written informed consent because of the study’s retrospective design and the use of data from administrative databases, thus, involving minimal risk to study participants.

## Results

### Incidence of IPD by age group and vaccine serotype

Between July 2008 and June 2016, 5324 cases of IPD were identified. A decreasing trend of annual IPD incidence was significant across all age groups (*p* values for trend in each age group < 0.05). Before the implementation of national PCV13 vaccination programs in children in 2013, the highest incidence of IPD in children ≤5 years occurred in the year of July 2010–June 2011, and a decrease from a peak of 21.23 cases per 100, 000 inhabitants during July 2010–June 2011 to 6.14 per 100,000 inhabitants during July 2015–June 2016 was determined (Table 1). A comparison of the IPD incidence of July 2013–June 2016 and July 2008–June 2013 showed reductions of 73% (95% CI: 68–77%) and 60% (95% CI: 50–68%) in PCV13 VT and serotype 19A, respectively. Non-PCV13 serotype (non-PCV13 ST) IPD increased from 1.88 per 100,000 inhabitants during July 2008–June 2013 to 3.38 per 100,000 inhabitants during July 2013–June 2016 (RR=1.80, 95% CI: 1.39–2.32). The IPD incidence among individuals 6–64 years who were not covered by national pneumococcal vaccination programs was lower than in other age groups. Reductions in PCV13 VT and PPV23 VT of 29% (95% CI: 20–36%) and 25% (95% CI: 17–33%), respectively, were determined when the IPD incidence of July 2013–June 2016 was compared with that of July 2008–June 2013. The incidence of non-PCV13 ST IPD increased from 0.41 per 100,000 inhabitants during July 2008–June 2013 to 0.52 during July 2013–June 2016 (RR = 1.27, 95% CI: 1.09–1.47). For the age group 65–74 years, a comparison of the IPD incidence during the periods July 2013–June 2016 and July 2008–June 2013 showed reductions in PCV13 VT and PPV23 VT of 43% (95% CI: 31–53%) and 40% (95% CI: 28–50%), respectively, together with a 38% (95% CI: 6–79%) increase in non-PCV13 ST IPD. Among adults ≥75 years, a 44% (95% CI: 9–65%) reduction of PPV23-non PCV13 VT IPD, a 36% (95% CI: 25–45%) reduction of PCV13 VT, and a 36% (95% CI: 26–45%) reduction of PPV23 VT, occurred based on a comparison of July 2013–June 2016 with July 2008–June 2013. Such decline of IPD in adults ≥75 years after the introduction of PCV13 is likely due to a combination of direct effects from PPV23 and indirect effects from PCV13. The serotype percentage of PPV23-non PCV13 VT among the non-PCV13 ST was 37.8, 41.7, 36.4, 19.5, 13.5, 25.7, 9.5, and 15.7%, respectively, in adults ≥75 years between July 2008–June 2009 and July 2015–June 2016 (Supplementary Table 1).

**Table 1:** Annual incidence (per 100,000 inhabitants) of IPD in Taiwan from July 2008 to June 2016 and a comparison of the incidence during July 2008–June 2009 vs. that during July 2015–June 2016 (*n* = 5324)

Age Group	Serotype	2008–2009	2009–2010	2010–2011	2011–2012	2012–2013	2013–2014	2014–2015	2015–2016	Trend test *p* value	July 2008–June 2013 incidence	July 2008–June 2013 incidence	2013–2016 vs. 2008–2013 RR	(95% CI)
≤5		*n* = 167	*n* = 175	*n* = 249	*n* = 206	*n* = 180	*n* = 101	*n* = 87	*n* = 75		*n* = 977	*n* = 263		
	Total cases	13.32	14.33	21.23	17.70	15.13	8.51	7.25	6.14	<.0001	16.28	7.29	0.45	(0.39–0.51)
	PCV13 VT	12.20	12.94	19.53	15.46	12.10	5.90	3.00	2.86	<.0001	14.40	3.91	0.27	(0.23–0.32)
	PPV23 VT	11.80	12.12	18.51	14.77	11.93	5.48	3.41	3.19	<.0001	13.78	4.02	0.29	(0.24–0.35)
	PPV23-non PCV13 VT	0.24	0.08	0.09	0.17	0.42	0.25	0.58	0.49	0.013	0.20	0.44	2.22	(1.05–4.69)
	Non-PCV13	1.12	1.39	1.71	2.23	3.03	2.61	4.25	3.27	<.0001	1.88	3.38	1.80	(1.39–2.32)
	19A	2.39	4.26	8.70	10.05	8.99	4.13	2.00	2.13	0.002	6.80	2.74	0.40	(0.32–0.50)
6–64		*n* = 300	*n* = 268	*n* = 322	*n* = 276	*n* = 279	*n* = 270	*n* = 207	*n* = 272		*n* = 1445	*n* = 749		
	Total cases	1.55	1.38	1.65	1.41	1.43	1.39	1.07	1.41	0.003	1.48	1.29	0.87	(0.79–0.95)
	PCV13 VT	1.13	0.98	1.31	1.02	0.93	0.94	0.55	0.81	<.0001	1.08	0.77	0.71	(0.64–0.80)
	PPV23 VT	1.15	1.04	1.33	1.02	0.95	0.97	0.61	0.89	<.0001	1.10	0.82	0.75	(0.67–0.83)
	PPV23-non PCV13 VT	0.06	0.10	0.08	0.09	0.13	0.09	0.09	0.12	0.115	0.09	0.10	1.08	(0.78–1.50)
	Non-PCV13	0.42	0.40	0.34	0.39	0.50	0.44	0.51	0.60	< 0.001	0.41	0.52	1.27	(1.09–1.47)
	19A	0.03	0.08	0.18	0.17	0.21	0.26	0.07	0.20	<.0001	0.13	0.18	1.31	(1.02–1.70)
65–74		*n* = 102	*n* = 109	*n* = 100	*n* = 88	*n* = 86	*n* = 79	*n* = 89	*n* = 83		*n* = 485	*n* = 251		
	Total cases	7.43	7.83	7.22	6.31	6.01	5.31	5.69	5.03	<.0001	6.95	5.34	0.77	(0.66–0.89)
	PCV13 VT	5.39	6.18	5.56	4.80	4.47	3.56	2.81	2.72	<.0001	5.28	3.02	0.57	(0.47–0.69)
	PPV23 VT	6.05	6.54	6.06	4.88	4.61	3.90	3.39	2.91	<.0001	5.62	3.38	0.60	(0.50–0.72)
	PPV23-non PCV13 VT	0.73	0.57	0.94	0.14	0.42	0.40	0.64	0.24	0.067	0.56	0.43	0.76	(0.44–1.30)
	Non-PCV13	2.04	1.65	1.66	1.51	1.54	1.75	2.88	2.30	0.064	1.68	2.32	1.38	(1.06–1.79)
	19A	0.15	0.36	0.36	0.43	0.84	0.54	0.70	0.54	0.026	0.43	0.60	1.38	(0.83–2.32)
≥ 75		*n* = 166	*n* = 138	*n* = 151	*n* = 162	*n* = 147	*n* = 125	n = 147	*n* = 118		*n* = 764	*n* = 390		
	Total cases	16.11	12.94	13.70	14.29	12.57	10.37	11.82	9.17	<.0001	13.89	10.44	0.75	(0.67–0.85)
	PCV13 VT	12.52	9.57	9.71	10.67	8.12	7.46	6.75	5.21	<.0001	10.07	6.45	0.64	(0.55–0.75)
	PPV23 VT	13.69	10.88	10.52	10.58	8.29	7.96	7.07	5.67	<.0001	10.72	6.88	0.64	(0.55–0.74)
	PPV23-non PCV13 VT	1.36	1.41	1.45	0.71	0.60	0.75	0.48	0.62	0.001	1.09	0.62	0.56	(0.35–0.91)
	Non-PCV13	3.59	3.38	3.99	3.62	4.45	2.90	5.06	3.96	0.253	3.82	3.99	1.04	(0.85–1.29)
	19A	0.29	0.19	0.27	0.71	1.11	1.08	0.96	1.09	0.000	0.53	1.04	1.98	(1.22–3.20)
all ages		*n* = 735	*n* = 690	*n* = 822	*n* = 732	*n* = 692	*n* = 575	*n* = 530	*n* = 548		*n* = 3671	*n* = 1653		
	Total cases	3.19	2.98	3.55	3.15	2.97	2.46	2.26	2.33	<.0001	3.17	2.35	0.74	(0.70–0.79)
	PCV13 VT	2.50	2.32	2.89	2.45	2.08	1.70	1.16	1.29	<.0001	2.45	1.38	0.56	(0.53–0.61)
	PPV23 VT	2.58	2.41	2.92	2.41	2.10	1.75	1.28	1.41	<.0001	2.49	1.48	0.60	(0.55–0.64)
	PPV23-non PCV13 VT	0.16	0.19	0.20	0.13	0.19	0.15	0.17	0.17	0.813	0.17	0.17	0.96	(0.77–1.21)
	Non-PCV13	0.69	0.67	0.66	0.71	0.89	0.76	1.11	1.04	<.0001	0.72	0.97	1.34	(1.21–1.48)
	19A	0.18	0.32	0.63	0.71	0.74	0.52	0.26	0.37	0.220	0.52	0.38	0.74	(0.64–0.86)

*IPD* invasive pneumococcal disease, *VT* vaccine type, *RR* rate ratio, *CI* confidence interval, *PCV13* thirteen-valent pneumococcal conjugate vaccine, *PPV23* 23-valent pneumococcal polysaccharide vaccine, *PCV13 VT* serotypes of 1, 3, 4, 5, 6A, 6B, 7F, 9 V, 14, 18C, 19A, 19F, and 23F, *PPV23 VT* serotypes of 1, 2, 3, 4, 5, 6B, 7F, 8, 9 N, 9 V, 10A, 11A, 12F, 14, 15B,17F, 18C, 19A, 19F, 20, 22F, 23F and 33F, *PPV23-non PCV13 VT* 11 serotypes that included in PPV23 but not in PCV13, *Non-PCV13* serotypes not found in PCV13 regardless of their relationship to PPV23 serotypes

### Characteristics of IPD in adults ≥ 75 years

**Fig. 1:**
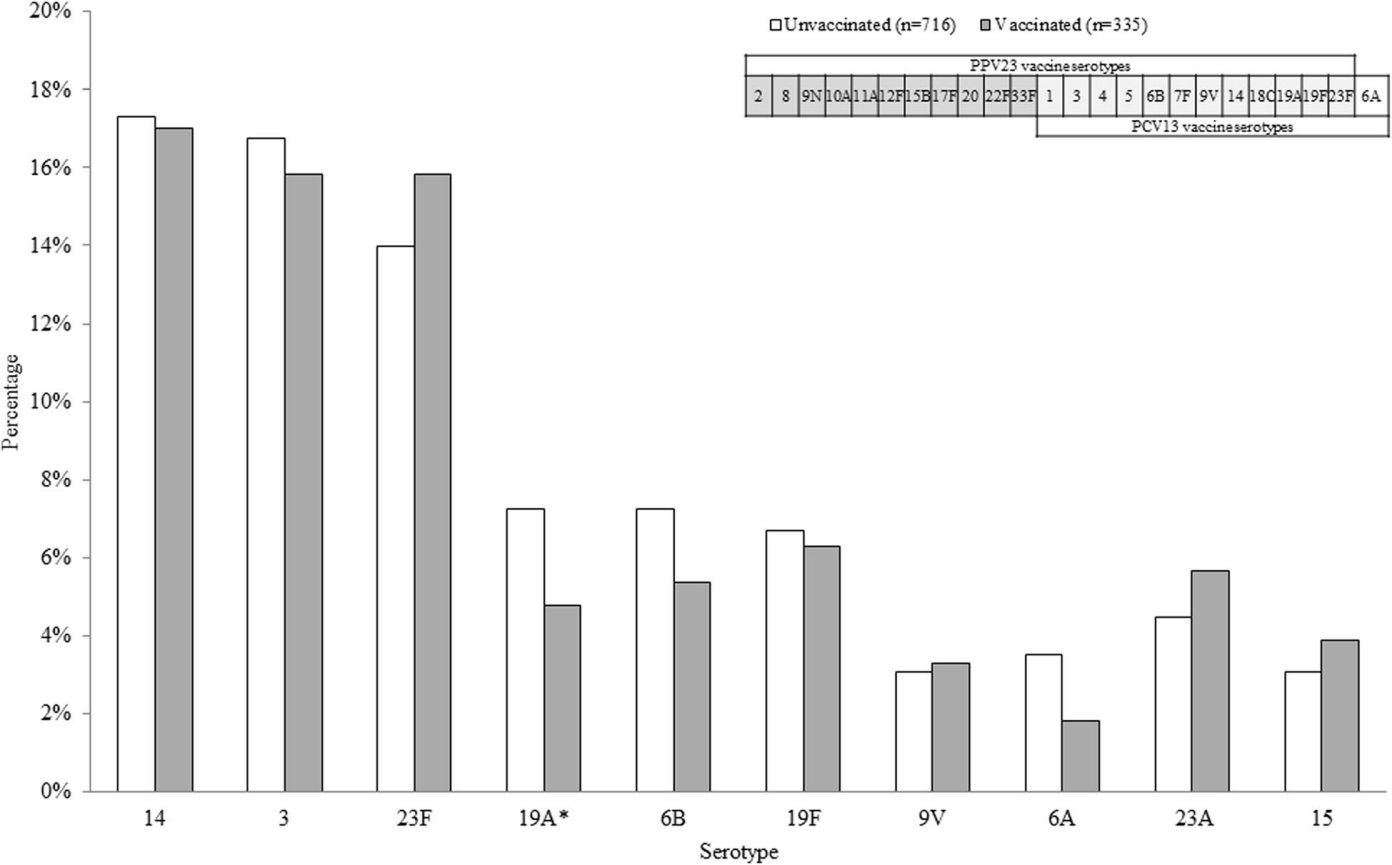
Distribution of the serotypes identified in at least 30 cases of invasive pneumococcal disease in patients aged 75 years and over, stratified by PPV23 vaccination status, Taiwan, July 2008–June 2016, *n* = 1051. The Y axis indicates the percentage of serotype-specific IPD cases among PPV23 vaccinated and unvaccinated IPD patients, respectively. Among the IPD patients, serotypes 14, 3, 23F, 19A, 6B, 19F, and 9 V were included in both PPV23 and PCV13. Serotype 6A is included in PCV13 but not in PPV23. Serotypes 23A and 15 (excluded 15B) are included in neither PPV23 nor PCV13. *: Serotype 19A differed significantly between vaccinated and unvaccinated individuals (*p* < 0.05)

The incidence of IPD in adults ≥75 years of age varied from 16.11 per 100,000 inhabitants during July 2008–June 2009 to 9.17 per 100,000 inhabitants during July 2015–June 2016 (*p* for trend <.0001). Table 2 shows the characteristics of the 1154 IPD patients who were ≥75 years old, the target population of the national PPV23 program. Among them, IPD affected more men (782 cases, 67.8%) than women, with pneumonia as the main clinical manifestation (776 cases, 67.2%). PPV23 had been administered to 378 individuals (32.8%) and 591 (51.2%) had HRMC. Serotyping was available for 1051 patients (91.1%). The proportion of PPV23 serotypes was 65.9% in the vaccinated and 77.1% in the unvaccinated. Vaccinated patients were older at the age of IPD onset (*p* < 0.05), presented with more HRMCs (*p* < 0.05), and had a lower proportion of PPV23-serotype IPD (*p* < 0.001) than unvaccinated patients. As shown in Fig. 1, the most common pneumococcal serotypes identified in adults ≥75 years were: 14 (181 patients, 17.2%), 3 (173 patients, 16.5%), 23F (153 patients, 14.6%), 6B (70 patients, 6.7%), 19F (69 patients, 6.6%), and 19A (68 patients, 6.5%), respectively. Of these, the only serotype that significantly differed between vaccinated and unvaccinated IPD patients was 19A (*p* < 0.05).

**Table 2:** Characteristics of IPD in patients ≥75 years according to PPV23 status, July 2008 to June 2016 (*n* = 1154)

	Total	%	Unvaccinated	%	Vaccinated	%	*p* value
Total IPD cases	1154	100	776	100	378	100	
Total IPD case serotyped	1051	91.1	716	92.3	335	88.6	0.042
Gender							
Female	372	32.2	258	33.2	114	30.2	0.292
Male	782	67.8	518	66.8	264	69.8	0.292
Onset age							
Median (min~max)	83.1	(75.0~106.6)	82.8	(75.0~106.6)	83.5	(75.1~99.8)	0.002
75–84	728	63.1	499	64.3	229	60.6	0.219
85+	426	36.9	277	35.7	149	39.4	0.219
Clinical manifestation							
Sepsis	621	53.8	417	53.7	204	54.0	0.941
Pneumonia	776	67.2	513	66.1	263	69.6	0.239
Meningitis	10	0.9	5	0.6	5	1.3	0.311
Others	1081	93.7	720	92.8	361	95.5	0.108
Death within 30 days of IPD onset							
N	432	37.4	287	37.0	145	38.4	0.651
Age, Medium (min~max)	83.6	(75.0~100.3)	83.6	(75.0~100.3)	84.1	(75.1~98.2)	0.330
Days between onset to notification							
Median (min~max)	5	(0.0~64.0)	5	(0.0~64.0)	5	(0~40.0)	0.710
Onset season							
2008–2009	166	14.4	130	16.8	36	9.5	0.001
2009–2010	138	12.0	100	12.9	38	10.1	0.164
2010–2011	151	13.1	107	13.8	44	11.6	0.310
2011–2012	162	14.0	107	13.8	55	14.6	0.727
2012–2013	147	12.7	101	13.0	46	12.2	0.686
2013–2014	125	10.8	81	10.4	44	11.6	0.538
2014–2015	147	12.7	83	10.7	64	16.9	0.003
2015–2016	118	10.2	67	8.6	51	13.5	0.011
Total IPD cases	1154	100	776	100	378	100	
High risk medical conditions (HRMC)							
With HRMC	591	51.2	378	48.7	213	56.3	0.019
Immunodeficiency/cancer	240	20.8	152	19.6	88	23.3	0.147
COPD	214	18.5	133	17.1	81	21.4	0.079
Congenital heart disease	6	0.5	3	0.4	3	0.8	0.400
Splectomy/Asplenism	2	0.2	1	0.1	1	0.3	0.548
Others	189	16.4	125	16.1	64	16.9	0.723
Serotypes							
PCV13 VT	795	68.9	554	71.4	241	63.8	0.009
PPV23 VT	847	73.4	598	77.1	249	65.9	<.0001
PPV23-non PCV13 VT	83	7.2	69	8.9	14	3.7	0.001

*IPD* invasive pneumococcal disease, *COPD* chronic obstructive pulmonary diseases, *VT* vaccine type, *PCV13* thirteen-valent pneumococcal conjugate vaccine, *PPV23* 23-valent pneumococcal polysaccharide vaccine, *PPV23-non PCV13 VT* 11 serotypes that included in PPV23 but not in PCV13, that is serotypes of 2, 8, 9 N, 10A, 11A, 12F, 15B, 17F, 20, 22F, and 33F

### Vaccine uptake

Supplementary Table 2 shows the accumulated vaccine coverage of the population vaccinated with the PPV23 vaccine by June 30, 2016, according to the age groups 65–74, 75–84 and ≥85 years. The data are nationally and regionally based and were calculated from NIIS data. National PPV23 coverage for people ≥75 years of age was 41.9%. Vaccine coverage was higher in the seven counties/cities than in other counties/cities for the age groups 65–74 and ≥75 years. In order to validate the PPV23 vaccination records for elderly adults in NIIS, an estimation of 80.6% of 853,460 doses of PPV23 procurement by the government between July 2008 to June 2016 was registered with vaccinee information identified in the NIIS database, including 570,665 and 117,295 individuals immunized with PPV23 vaccination at age of ≧75 years and 65–74 years, respectively. The missing PPV23 records could be possibly explained by the doses that have not been used, doses that have been wasted, or a truly unregistered PPV23 vaccination records.

### Vaccine effectiveness

PPV23VE in adults ≥75 years as estimated by the screening method is shown in Table 3. The sex- and age-adjusted VE was 33.9% (95% CI: 25.2–41.5%) in preventing all-serotype IPD, 32.5% (95% CI: 17.5–44.7%) in preventing death within 30 days of IPD onset, 43.4% (95% CI: 34.4–51.2%) in preventing IPD caused by PPV23 VT, 72.6% (95% CI: 51.4–84.6%) in preventing IPD caused by PPV23-non PCV13 VT, and 39.6% (95% CI: 29.5–48.2%) in preventing IPD caused by PCV13 (excluding 6A) VT.

**Table 3:** Vaccine effectiveness of PPV23 in adults ≥75 years, estimated by the screening method, July 2008 to June 2016

IPD	VE	(95% CI)
All serotypes		
Crude VE	32.4%	(23.6 to 40.2)
Adjusted by gender	33.2%	(24.4 to 40.9)
Adjusted by age and gender	33.9%	(25.2 to 41.5)
Death within 30 days of IPD onset		
Crude VE	29.9%	(14.4 to 42.6)
Adjusted by gender	30.8%	(15.5 to 43.4)
Adjusted by age and gender	32.5%	(17.5 to 44.7)
PPV23 VT		
Crude VE	42.2%	(33.0 to 50.2)
Adjusted by gender	42.9%	(33.8 to 50.7)
Adjusted by age and gender	43.4%	(34.4 to 51.2)
PPV23-non PCV13 VT		
Crude VE	71.8%	(50.0 to 84.2)
Adjusted by gender	72.1%	(50.5 to 84.3)
Adjusted by age and gender	72.6%	(51.4 to 84.6)
PCV13 (without 6A) VT		
Crude VE	38.4%	(28.1 to 47.1)
Adjusted by gender	39.1%	(29.0 to 47.8)
Adjusted by age and gender	39.6%	(29.5 to 48.2)

*IPD* invasive pneumococcal disease, *VE* vaccine effectiveness, *CI* confidence interval, *VT* vaccine type, *PPV23* 23-valent pneumococcal polysaccharide vaccine, *PCV13* thirteen-valent pneumococcal conjugate vaccine, *PPV23-non PCV13 VT* 11 serotypes that included in PPV23 but not in PCV13, that is serotypes of 2, 8, 9 N, 10A, 11A, 12F, 15B, 17F, 20, 22F, and 33F

The effectiveness of PPV23 as estimated by the indirect cohort method was 39.0% (95% CI: 15.5–55.9%; Table 4), which corresponded to a number needed to vaccinate of 13,173 (95% CI: 10,093–33,146) per PPV23-serotype IPD case prevented. VE was not significant in subgroups of patients ≥85 years (33.9%; 95% CI: − 15.2 to 62.1) and in patients with HRMCs (20.4%; 95% CI: − 24.5 to 49.1). VE increased when only 11 serotypes included in PPV23 but not in PCV13 were considered. According to the indirect cohort method (Table 4), VE in preventing PPV23-non PCV13 VT was 71.5% (95% CI: 44.2–85.4%), which was similar to the VE estimated by the screening method (Table 3). For the PPV23 serotypes identified in at least 30 cases, a significant VE was determined for serotypes 15B, 19A, 6B, 3 and 14 but not for serotypes 23F and 19F (Table 4). Supplementary Table 3 shows VE against IPD from July 2008 to June 2012, by excluding the PCV13 program period, and a higher point estimate of VE was observed for PPV23 VT (VE=55.1, 95% CI: 27.2–72.3%) and PPV23-non PCV13 VT (VE=79.8, 95% CI: 47.2–92.3%), respectively, while comparing with VE estimated against the same serotypes during the period July 2018–June 2016.

**Table 4:** Effectiveness of PPV23 in patients ≥75 years of age, estimated by the indirect cohort (Broome) method, July 2008 to June 2016 (*n *= 1051)

IPD	Cases	Controls	Crude VE (%)	95%CI	Adjusted VE (%)	95%CI
Serotypes PPV23	847	204	42.9	(21.7 to 58.3)	39.0^a^	(15.5 to 55.9)
75–84	540	133	44.9	(18.5 to 62.8)	42.4^b^	(13.7 to 61.6)
85+	307	71	39.5	(−2.5 to 64.3)	33.9 ^b^	(−15.2 to 62.1)
Female	263	82	46.1	(9.5 to 68.0)	38.7^c^	(−4.9 to 64.2)
Male	584	122	42.5	(14.3 to 61.4)	38.0^c^	(6.4 to 59.0)
Without HRMC	390	95	57.6	(32.3 to 73.4)	56.5^d^	(29.6 to 73.1)
With HRMC	425	108	27.5	(−11.7 to 52.9)	20.4^d^	(−24.5 to 49.1)
> 5 years after vaccination	668	143	44.7	(9.1 to 66.4)	15.5^a^	(−47.1 to 51.4)
≤ 5 years after vaccination	777	179	42.1	(17.7 to 59.2)	44.9^a^	(20.8 to 61.7)
Serotypes PPV23-non PCV13	83	204	72.2	(47.3 to 85.3)	71.5^a^	(44.2 to 85.4)
Serotype PCV13 (without 6A)	764	204	39.0	(16.3 o 55.6)	35.3^a^	(10.1 to 53.4)
serotype 3	173	204	39.4	(7.2 to 60.4)	36.8^a^	(0.4 to 59.9)
Serotype14	181	204	36.9	(4.1 to 58.5)	38.8^a^	(4.1 to 61.0)
serotype 23F	153	204	27.3	(−12.2 to 52.9)	11.3^a^	(−42.1 to 44.7)
serotype 19F	69	204	40.0	(−7.6 to 66.5)	34.9^a^	(−21.3 to 65.1)
serotype 19A	68	204	57.8	(21.1 to 77.4)	56.3^a^	(16.1 to 77.2)
serotype 6B	70	204	52.5	(13.1 to 74.0)	51.9^a^	(8.1 to 74.8)
serotype 15B	23	204	79.4	(28.5 to 94.1)	80.6^a^	(28.6 to 94.7)
serotype 9 V	33	204	31.4	(−49.0 to 68.4)	31.3^a^	(−60.4 to 70.6)

*VE* vaccine effectiveness, *CI* confidence interval, *HRMC* high-risk medical conditions, *PPV23* 23-valent pneumococcal polysaccharide vaccine, *PCV13* thirteen-valent pneumococcal conjugate vaccine. Serotypes PPV23-non PCV13 VT: 11 serotypes that included in PPV23 but not in PCV13, that is serotypes of 2, 8, 9 N, 10A, 11A, 12F, 15B, 17F, 20, 22F, and 33F

^a^Adjusted for age group, sex, presence of HRMC, and onset year; ^b^Adjusted for sex, presence of HRMC, and onset year; ^c^Adjusted for age group, presence of HRMC, and onset year; ^d^Adjusted for age group, sex, and onset year

### Differential vaccine effectiveness of PPV23 over time

We also assessed VE according to different intervals after vaccination. The adjusted PPV23VE in adults ≥75 years according to the indirect cohort method was 44.9% (95% CI: 20.8–61.7) within 5 years of vaccination and 15.5% (95% CI: − 47.1 to 51.4) thereafter (Table 4). The adjusted PPV23VE in adults ≥75 years of age was 73.7% (39.0–88.7) when ≤1 year had elapsed, 64.4% (29.2–82.0) when > 1 year but ≤ 2 years had elapsed, 48.9% (95% CI: − 1.6 to 74.3) when > 2 years but ≤ 3 years had elapsed, 19.2% (95% CI: − 61.3 to 59.5) when > 3 years but ≤ 4 years had elapsed, and − 3.4% ((95% CI: − 144.9 to 56.3) when > 4 years but ≤ 5 years had elapsed (Table 5). The wide interval for the > 4 and ≤ 5 years might be explained by the smaller sample size, compared to other categories of time since vaccination.

**Table 5:** Effectiveness of PPV23 in patients ≥75 years of age at different intervals after vaccination, estimated by the indirect cohort (Broome) method, July 2008 to June 2016 (*n *= 1051)

Time since PPV23 vaccination	Cases	Controls	Crude VE (%)	95%CI	Adjusted VE (%)	95%CI
No vaccination	598	118	Ref.		Ref.	
≤ 1 years	29	11	48.0	(−7.0 to 74.7)	73.7	(39.0 to 88.7)
> 1 and ≤ 2 years	41	15	46.1	(−0.6 to 71.1)	64.4	(29.2 to 82.0)
> 2 and ≤ 3 years	37	14	47.9	(0.5 to 72.7)	48.9	(−1.6 to 74.3)
> 3 and ≤ 4 years	46	13	30.2	(−33.3 to 63.4)	19.2	(−61.3 to 59.5)
> 4 and ≤ 5 years	26	8	35.9	(−45.1 to 71.7)	−3.4	(−144.9 to 56.3)
> 5 years	70	25	44.7	(9.1 to 66.4)	15.5	(−47.1 to 51.4)

*PPV23* 23-valent pneumococcal polysaccharide vaccine, *Ref* reference, *VE* vaccine effectiveness, *CI* confidence interval

## Discussion

For countries with long-term laboratory-based systems for monitoring IPD, linking IPD surveillance with valid pneumococcal immunization records facilitates the evaluation of vaccination programs pre- and post-implantation from a public health perspective [1]. In determinations of VE, the results of effectiveness studies might be at risk of bias due to the impact of patient health and the short study period, as was the case in a previously published nationwide study of PPV23 effectiveness in Taiwan. In contrast, our study used data from subsequent years in similar adult populations and drew on different observational methodologies [9, 16]. The results showed that with respect to IPD and 30-day fatality, PPV23VE was lower than expected when the study period was extended to 8 years. Based on observational studies, the estimated PPV23VE against IPD in older adults or adults with conditions associated with an increased risk of IPD was 27–76% [9, 11, 13, 16, 24, 26, 27]. Previously reported estimates of VE have differed, most likely because of the different methods used and the different study periods in the estimations [14, 27]. The point estimate of PPV23VE in this study was within the 95% CI of the estimated VE reported in a Cochrane review of non-random controlled trials in adults (VE = 52, 95% CI: 39–63%) [13].

The decline in IPD among adults ≥ 75 years from pre- to post-PCV13 immunization era is likely due to a combination of direct effects from PPV23 and indirect effects from PCV13, which are epidemiologically challenging to tease apart. Therefore, we applied the indirect cohort method to evaluate PPV23VE during the period July 2008–June 2012 when national PCV13 program was not introduced in children. PPV23VE for elderly seemed higher in the period of July 2008–June 2012 (VE=55.1, 95% CI: 27.2–72.3%) than in the period of July 2008–June 2016 (VE=39.0, 95% CI: 15.5–50.9%). We speculated that as the indirect protection emerged following PCV13 introduction, PPV23 unvaccinated elderly could benefit from lowering their risk of acquiring IPD caused by 12 serotypes common to PCV13 and PPV23, which might bias the PPV23VE estimation to be lower. On the other hand, VE waning since time of vaccination should possibly be considered.

In addition, in our study, PPV23VE was higher against IPD caused by 11 serotypes that included in PPV23 but not in PCV13 (PPV23-non PCV13 VT) than against PPV23 serotype-IPD, as also found in a Spanish study [10]. Such findings were observed using either the screening method or the indirect cohort method. This difference may be due to a differential effectiveness against different serotypes [10, 28]. However, other possible causes remain to be explored in future studies [10, 23, 28, 29].

The validity of VE estimation using the screening method could be influenced by the completeness of PPV23 immunization recorded in the NIIS. Although the accurate PPV23 vaccination coverage for IPD cases and elderly population might be greater than 32.8 and 41.9%, respectively, it may not result in very low or very high *Pp* and *Pc* to bias VE estimation [30, 31]. PPV23VE estimates by the indirect cohort method would possibly bias if the PPV23 vaccinated and unvaccinated individuals are not at the same risk of non-PPV23 ST infections [10, 23]. Unlike PCV-induced serotype replacement, PPV23 would not drive the increase of non-PPV23 ST among vaccinated and unvaccinated elderly.

The adjusted VE determined by the screening method was slightly higher than the estimated VE obtained using the Broome method, perhaps because the screening method does not allow for the control of confounding factors, which can result in an overestimation of VE [21]. Moreover, the study population applied in the Broome method consisted of all patients who had developed IPD (case-case comparison approach) [32], in whom the proportion of underlying disease may have been higher than in community-dwelling elderly. Differences in the immune responses to vaccination of the case-case population compared to the community-dwelling elderly population may lead to a lower VE estimate [16, 33].

Following the 2006 recommendation by the WHO of a routine PCV-based immunization program in children, the IPD incidence caused by PCV VT gradually declined not only in vaccinated (direct protection) but also in unvaccinated (indirect protection) age groups [3, 34, 35]. From before (July 2008–June 2013) to after (July 2013–June 2016) the implementation of a children’s PCV13 program in Taiwan, the highest age-specific annual IPD incidence shifted from age 2–4 years to ≥ 75 years, with a decreasing trend in the incidence of IPD related to PCV13 VT across all age groups. Previous studies reported an increase in IPD, due to non-PCV serotypes or serotype replacement, roughly 3–4 years after the introduction of a PCV program [36–38]. The increasing trend in the incidence of non-PCV13 ST IPD determined in this study involved all age groups except adults ≥75 years, i.e., the target population of the national PPV23 program. Whether serotype replacement occurs over different time intervals in different age groups or is influenced by the different serotype distributions among age groups, especially for those covered by the PPV23 vaccination, remains to be investigated. Long-term surveillance of IPD, nasal carriage, and non-bacteremic pneumococcal pneumonia will be crucial in the monitoring of serotype replacement and in ascertaining whether pneumococcal vaccines offer direct or indirect protection against the incidence of disease over the long term [36].

Vaccine protection at the population level can be rapidly estimated using the screening method whereas the duration of PPV23 protection at an individual level could be estimated by a stratified analysis of time since vaccination using the indirect cohort method [10, 11, 21]. In this study, the change in PPV23VE since the time of vaccination, measured at an interval of every 1-year between IPD onset and the date of vaccination, could be considered a diagnostic indication of waning VE [10, 15, 39]. The wide confidence interval and biased point estimate of VE in the subgroup of the interval of > 4 and ≤ 5 years could be possibly explained by the small sample size [40]. A study from England and Wales reported a PPV23VE of 48% against IPD within 2 years of vaccination for adults ≥65 years, as determined by the indirect cohort method, but VE waned and became insignificant beyond 5 years [28]. In the effectiveness of pneumococcal vaccination against community-acquired pneumonia, acute myocardial infarction and stroke (CAPAMIS) study, effectiveness estimates became higher and significant after patients vaccinated > 60 months previously were excluded [41]. The differential VE against PPV23-serotype IPD observed over time in our study was in agreement with a previous report but the lack of significance of the 2- to 5-year interval may have been due to the lower statistical power resulting from stratification of the variable “time since vaccination” [10].

PPV23 is a T-cell-independent vaccine that lacks a mechanism for long-term boosting of the immune response [42]. While PPV23 revaccination may result in a significant and sustained antibody responses in adults, including the elderly [43, 44], it is recommended only for those with an increased risk of IPD and no sooner than 5 years after the first dose [45]. However, insufficient data regarding clinical benefit, the degree and duration of protection, and safety have hindered a routine recommendation of revaccination [46]. In the USA, as in Taiwan, a single dose of PPV23 is recommended for all adults ≥65 years of age regardless of the previous history of PPV23 [3, 46]. In the elderly and in risk groups in middle-high income countries, PCV13 and PPV23 immunization is recommended to confer better and longer protection [42, 47]. In fact, in many countries, PPV23 is recommended as an effective and/or cost-effective vaccine covering a broad array of the serotypes implicated in IPD in older adults [10, 45, 48].

Our study had several limitations beyond its observational design and the residual confounders. First, although the coverage of hospitals enrolled in IPD surveillance in Taiwan should have been 100%, detection bias may have occurred for cases identified using this passive surveillance system, thereby affecting the representativeness of the data [2, 17]. Second, PPV23 vaccine records in the NIIS database were prospectively collected after the endorsement of a publicly funded PPV vaccination program for elderly individuals. Information on missing vaccination status was not determined by the occurrence of IPD outcome. Therefore, the probability of misclassification of vaccinated to be unvaccinated might be non-differential between cases and controls in the indirect cohort method. Third, IPD surveillance has been started with the implementation of the national PPV23 vaccination program in the elderly. We could not evaluate the impact of PPV23 program from pre- to post-PPV23 vaccination program by changes of IPD incidence. Fourth, influenza vaccination status is an important covariate that should be considered in evaluations of PPV23VE in the elderly [49]. However, the NIIS database contains limited annual individual influenza vaccine records and it was not possible to determine seasonal influenza status as covariate information for the elderly population in our study.

## Conclusions

In conclusion, our results demonstrated a decrease in IPD incidence among all age groups across eight seasons covering the pre- to post-national PCV13 immunization era in Taiwan, accompanied by an increased trend of non-PCV13 ST IPD. The decline in IPD among adults ≥ 75 years is likely due to a combination of direct effects from PPV23 and indirect effects from PCV13. Using the indirect cohort method, PPV23 did confer moderate protection against PPV23-serotype IPD in adults ≥75 years before national PCV13 immunization program implemented in children.

## Supplementary Information

**Additional file 1: Supplementary Table 1.** Serotype percentage of IPD in Taiwan from July 2008 to June 2016, stratified by age group.

**Additional file 2: Supplementary Table 2.** Accumulated coverage of PPV23 in older adults, stratified by counties and age groups in Taiwan.

**Additional file 3: Supplementary Table 3.** Effectiveness of PPV23 in patients ≥75 years of age, estimated by the indirect cohort (Broome) method, July 2008 to June 2012 (*n *= 581).

## Data Availability

The datasets used and analyzed during the current study are not publicly available due to it contains individual data and maintained by the public health authorities but are available from the corresponding author on reasonable request.

## Abbreviations

IPD: Invasive pneumococcal disease
PCV: Pneumococcal conjugate vaccine
VE: Vaccine effectiveness
PPV23: 23-valent pneumococcal polysaccharide vaccine
PCV13: 13-valent PCV
WHO: World Health Organization
HRMC: High-risk medical condition
NIIS: National Immunization Information System
RR: Relative risk
*Pc*: Proportion of cases who have been vaccinated
*Pp*: Proportion of the target population who have been vaccinated
VT: Vaccine type
ST: Serotype
CAPAMIS: Community-acquired pneumonia, acute myocardial infarction and stroke
