# Supplementary material for: Application of the screening and indirect cohort methods to evaluate the effectiveness of pneumococcal vaccination program in adults 75 years and older in Taiwan
Source: BMC Infect Dis. 2021 Jan 10;21:45. doi: 10.1186/s12879-020-05721-0 (PMC7798272; doi:10.1186/s12879-020-05721-0)
Supplement: Supplementary file 1 — Additional file 1: Supplementary Table 1. Serotype percentage of IPD in Taiwan from July 2008 to June 2016, stratified by age group. [file 12879_2020_5721_MOESM1_ESM.docx]

**Supplementary Table 1. Serotype percentage of IPD in Taiwan from July 2008 to June 2016, stratified by age group**

| Serotype Percentage | 2008-2009 | 2009-2010 | 2010-2011 | 2011-2012 | 2012-2013 | 2013-2014 | 2014-2015 | 2015-2016 |
| --- | --- | --- | --- | --- | --- | --- | --- | --- |
| ≤5 |  |  |  |  |  |  |  |  |
| non-PCV13/All IPD | 8.4% | 9.7% | 8.0% | 12.6% | 20.0% | 30.7% | 58.6% | 53.3% |
| non-PPV23/All IPD | 11.4% | 15.4% | 12.9% | 16.5% | 21.1% | 35.6% | 52.9% | 48.0% |
| non-VT/All IPD | 6.6% | 9.1% | 7.6% | 11.7% | 17.2% | 27.7% | 50.6% | 45.3% |
| PPV23-non PCV13 VT/All IPD | 1.8% | 0.6% | 0.4% | 1.0% | 2.8% | 3.0% | 8.0% | 8.0% |
| (PPV23-non PCV13 VT)/(non-PCV13) | 21.4% | 5.9% | 5.0% | 7.7% | 13.9% | 9.7% | 13.7% | 15.0% |
| 6－64 |  |  |  |  |  |  |  |  |
| non-PCV13/All IPD | 27.0% | 29.1% | 20.5% | 27.5% | 34.8% | 31.9% | 48.3% | 42.6% |
| non-PPV23/All IPD | 25.7% | 24.3% | 19.3% | 27.5% | 33.7% | 30.0% | 43.0% | 36.8% |
| non-VT/All IPD | 23.3% | 21.6% | 15.5% | 21.0% | 25.4% | 25.2% | 39.6% | 34.2% |
| PPV23-non PCV13 VT/All IPD | 3.7% | 7.5% | 5.0% | 6.5% | 9.3% | 6.7% | 8.7% | 8.5% |
| (PPV23-non PCV13 VT)/(non-PCV13) | 13.6% | 25.6% | 24.2% | 23.7% | 26.8% | 20.9% | 18.0% | 19.8% |
| 65－74 |  |  |  |  |  |  |  |  |
| non-PCV13/All IPD | 27.5% | 21.1% | 23.0% | 23.9% | 25.6% | 32.9% | 50.6% | 45.8% |
| non-PPV23/All IPD | 18.6% | 16.5% | 16.0% | 22.7% | 23.3% | 26.6% | 40.4% | 42.2% |
| non-VT/All IPD | 17.6% | 13.8% | 10.0% | 21.6% | 18.6% | 25.3% | 39.3% | 41.0% |
| PPV23-non PCV13 VT/All IPD | 9.8% | 7.3% | 13.0% | 2.3% | 7.0% | 7.6% | 11.2% | 4.8% |
| (PPV23-non PCV13 VT)/(non-PCV13) | 35.7% | 34.8% | 56.5% | 9.5% | 27.3% | 23.1% | 22.2% | 10.5% |

**Supplementary Table 1.** *Continued*

| Serotype Percentage | 2008-2009 | 2009-2010 | 2010-2011 | 2011-2012 | 2012-2013 | 2013-2014 | 2014-2015 | 2015-2016 |
| --- | --- | --- | --- | --- | --- | --- | --- | --- |
| ≥75 |  |  |  |  |  |  |  |  |
| non-PCV13/All IPD | 22.3% | 26.1% | 29.1% | 25.3% | 35.4% | 28.0% | 42.9% | 43.2% |
| non-PPV23/All IPD | 15.1% | 15.9% | 23.2% | 25.9% | 34.0% | 23.2% | 40.1% | 38.1% |
| non-VT/All IPD | 13.9% | 15.2% | 18.5% | 20.4% | 30.6% | 20.8% | 38.8% | 36.4% |
| PPV23-non PCV13 VT/All IPD | 8.4% | 10.9% | 10.6% | 4.9% | 4.8% | 7.2% | 4.1% | 6.8% |
| (PPV23-non PCV13 VT)/(non-PCV13) | 37.8% | 41.7% | 36.4% | 19.5% | 13.5% | 25.7% | 9.5% | 15.7% |

IPD: invasive pneumococcal disease; VT: vaccine type; RR: rate ratio; CI: confidence interval; PCV13: thirteen-valent pneumococcal conjugate vaccine; PPV23: 23-valent pneumococcal polysaccharide vaccine; PCV13 VT: serotypes of 1, 3, 4, 5, 6A, 6B, 7F, 9V, 14, 18C, 19A, 19F, and 23F; PPV23 VT: serotypes of 1, 2, 3, 4, 5, 6B, 7F, 8, 9N, 9V, 10A, 11A, 12F, 14, 15B,17F, 18C, 19A, 19F, 20, 22F, 23F and 33F; PPV23-non PCV13 VT: 11 serotypes that included in PPV23 but not in PCV13; Non-PCV13: serotypes not found in PCV13 regardless of their relationship to PPV23 serotypes. Non-VT: serotypes that included in neither PPV23 nor PCV13.
