# Supplementary material for: Application of the screening and indirect cohort methods to evaluate the effectiveness of pneumococcal vaccination program in adults 75 years and older in Taiwan
Source: BMC Infect Dis. 2021 Jan 10;21:45. doi: 10.1186/s12879-020-05721-0 (PMC7798272; doi:10.1186/s12879-020-05721-0)
Supplement: Supplementary file 2 — Additional file 2: Supplementary Table 2. Accumulated coverage of PPV23 in older adults, stratified by counties and age groups in Taiwan. [file 12879_2020_5721_MOESM2_ESM.docx]

**Supplementary Table 2. Accumulated coverage of PPV23 in older adults, stratified by counties and age groups in Taiwan**

| Age group (years) | 65‒74 |  | ≧75 | | |
| --- | --- | --- | --- | --- | --- |
|  |  |  | 75‒84 | ≧85 | Sum |
| All counties (nationally) | | |  |  |  |
| Overall | 7.2% |  | 39.7% | 46.5% | 41.9% |
| Male | 7.1% |  | 40.9% | 45.5% | 42.6% |
| Female | 7.3% |  | 38.6% | 47.6% | 41.3% |
| Seven counties that endorsed additional PPV23 vaccination program regionally^*^ | | | | | |
| Overall | 28.6% |  | 50.6% | 51.1% | 50.7% |
| Male | 27.9% |  | 51.6% | 48.7% | 50.7% |
| Female | 29.2% |  | 49.8% | 53.2% | 50.8% |
| Non-seven counties | |  |  |  |  |
| Overall | 1.0% |  | 36.2% | 45.3% | 39.3% |
| Male | 1.0% |  | 37.6% | 44.7% | 40.3% |
| Female | 0.9% |  | 35.2% | 46.0% | 38.4% |

PPV23: 23-valent pneumococcal vaccine;

^*^: Seven counties/cities endorsed the PPV23 vaccination program for adults 65‒74 years of age, including the cities of Taichung, Tainan, and Chiayi and the counties Tainan, Yunlin, Taichung, and Lianjiang.
