# Supplementary material for: Application of the screening and indirect cohort methods to evaluate the effectiveness of pneumococcal vaccination program in adults 75 years and older in Taiwan
Source: BMC Infect Dis. 2021 Jan 10;21:45. doi: 10.1186/s12879-020-05721-0 (PMC7798272; doi:10.1186/s12879-020-05721-0)
Supplement: Supplementary file 3 — Additional file 3: Supplementary Table 3. Effectiveness of PPV23 in patients ≥75 years of age, estimated by the indirect cohort (Broome) method, July 2008 to June 2012 (n = 581). [file 12879_2020_5721_MOESM3_ESM.docx]

**Supplementary Table 3. Effectiveness of PPV23 in patients ≥75 years of age as estimated using the indirect cohort (Broome) method, July 2008 to June 2012 (n = 581)**

|  | Cases | Controls | Crude VE (%) | 95%CI | Adjust VE (%) | 95%CI |
| --- | --- | --- | --- | --- | --- | --- |
| Serotypes PPV23 | 493 | 88 | 55.2 | (28.2 to 72.0) | 55.1^a^ | (27.2 to 72.3) |
| 75-84 | 334 | 62 | 60.5 | (30.9 to 77.4) | 59.8^b^ | (28.3 to 77.4) |
| 85+ | 159 | 26 | 38.6 | (-48.8 to 74.7) | 36.0^b^ | (-61.9 to 74.7) |
| Female | 146 | 30 | 69.0 | (28.7 to 86.5) | 68.6^c^ | (25.4 to 86.8) |
| Male | 347 | 58 | 48.1 | (7.9 to 70.8) | 44.6^c^ | (-0.5 to 69.4) |
| Without HRMC | 237 | 46 | 59.1 | (20.7 to 78.9) | 60.8^d^ | (22.8 to 80.1) |
| With HRMC | 226 | 41 | 54.9 | (10.5 to 77.2) | 50.9^d^ | (0.1 to 75.8) |
| Serotypes PPV23-non PCV13 | 53 | 88 | 79.0 | (48.4 to 91.5) | 79.8^a^ | (47.2 to 92.3) |
| Serotype PCV13 (without 6A) | 440 | 88 | 51.8 | (22.6 to 70.0) | 52.7^a^ | (22.9 to 70.9) |
| serotype 3 | 101 | 88 | 66.0 | (35.0 to 82.2) | 69.9^a^ | (39.6 to 85.0) |
| Serotype14 | 113 | 88 | 45.5 | (1.9 to 69.8) | 55.7^a^ | (15.8 to 76.7) |
| serotype 23F | 94 | 88 | 47.3 | (2.1 to 71.6) | 33.7^a^ | (-29.7 to 66.1) |
| serotype 19F | 43 | 88 | 33.5 | (-43.1 to 69.1) | 21.0^a^ | (-78.4 to 65.0) |
| serotype 6B | 43 | 88 | 58.2 | (4.7 to 81.7) | 58.8^a^ | (0.5 to 82.9) |

VE: vaccine effectiveness; CI: confidence interval; HRMC: high-risk medical conditions; PPV23: 23-valent pneumococcal polysaccharide vaccine; PCV13: thirteen-valent pneumococcal conjugate vaccine. Serotypes PPV23-non PCV13 VT: 11 serotypes that included in PPV23 but not in PCV13, that is serotypes of 2, 8, 9N, 10A, 11A, 12F, 15B, 17F, 20, 22F, and 33F.

^a^Adjusted for age group, sex, presence of HRMC, and onset year; ^b^Adjusted for sex, presence of HRMC, and onset year; ^c^Adjusted for age group, presence of HRMC, and onset year; ^d^Adjusted for age group, sex, and onset year.
